# *Pseudomonas aeruginosa* in children with cerebral palsy: a prospective study

**DOI:** 10.3389/fped.2023.1267345

**Published:** 2023-11-06

**Authors:** Katrien Romaen, Isabelle Van Ussel, Carolin Van Rossem, Sandra Kenis, Berten Ceulemans, Kim Van Hoorenbeeck, Stijn Verhulst

**Affiliations:** ^1^ Department of Pediatric Neurology, Antwerp University Hospital/University of Antwerp, Antwerp, Belgium; ^2^ Department of Pediatric Pulmonology, Antwerp University Hospital and Lab of Experimental Medicine and Pediatrics, University of Antwerp, Antwerp, Belgium

**Keywords:** *Pseudomonas aeruginosa*, cerebral palsy, respiratory infection, gram-negative bacteria, children

## Abstract

**Introduction:**

Children with cerebral palsy (CP) often present with chronic respiratory symptoms. *Pseudomonas aeruginosa* (PA), is a known pathogen associated with more severe respiratory disease. Preventive actions to eradicate this bacterium and to improve the respiratory condition of children with CP could be very valuable. Therefore, we assessed the prevalence of PA and its association with respiratory disease.

**Methods:**

Throat swabs were taken in children with CP, aged 0–18 years. Data from patient records were extracted from the electronic medical records. Follow-up of respiratory symptoms was done by the Liverpool respiratory symptom questionnaire (LRSQ) after 3 months.

**Results:**

A throat swab and a completed LRSQ after 3 months were received from 79 children with CP. Twenty-eight patients (35.4%) were found to have at least one positive respiratory culture. Only 4 patients (5.1%) were contaminated with PA. Gram negative bacteria were isolated in 21.5% of the positive throat swabs, *S. aureus* was found in 13.9%. Most pathogens were found in patients with higher GMFCS score (GMFCS IV and V). Results of the LRSQ showed that 52.1% of these patients reported having 1 cold in the past 3 months.

**Discussion:**

The prevalence of PA in our population of children with CP is low, gram-negative bacteria were most commonly found. The respiratory consequences of being colonized with these bacteria were limited. These results may have been affected by the COVID-19 pandemic. Further research is recommended.

## Introduction

Children with cerebral palsy (CP) often present with chronic respiratory symptoms. This is an important risk factor for increased morbidity and mortality in children with CP. *Pseudomonas aeruginosa* (PA), is a known pathogen associated with more severe respiratory disease. Due to its ability to change its phenotype, an infection with PA is often chronic and PA is very difficult to eradicate. CP is often accompanied by multisystem medical concerns like epilepsy, secondary musculoskeletal problems and impairment in cognition, communication, behavior, perception, motor control and sensation (1–4). The degree or severity of motor disability is classified by the Gross Motor Function Classification System (GMFCS), a higher score indicates increasing severity and lower life expectancy (1, 4). Patients with CP have a shorter life expectancy than the general population and the observed morbidity and mortality is especially linked to respiratory disease (1, 3–5). The impact of respiratory morbidity on the quality of life cannot be underestimated (1, 2, 4). The reasons for these respiratory complications are probably multifactorial. Risk factors for hospital admission include the severity of gross motor disfunction reflected by GMFCS score, epilepsy, axial hypotonia, limited shoulder movement, severe kyphoscoliosis, swallowing problems, gastroesophageal reflux disease (GERD), gastrostomy feeding and absence or impairment of spontaneous cough (1, 3). Other medical conditions also influence hospitalization rate, quality of life and life expectancy, including overt or silent aspiration, impaired mucociliary clearance, kyphoscoliosis, upper or lower airway obstruction and recurrent infections leading to bronchiectasis. One study showed that the presence of abnormal bacterial flora (including Pseudomonas and Klebsiella species) in children with CP who are critically ill occurred twice as frequent compared to those critically ill without CP (5).

From experience in children with cystic fibrosis, it is known that chronic colonization of the lower airways with PA is a risk factor for repeated lung infections, deterioration in lung function and shortened survival (6). It is also known that early therapy will prevent chronic colonization (6). Preventive actions to eradicate this bacterium and to improve the respiratory condition of children with CP could therefore be of interest. The prevalence and role of PA, however, is relatively unknown in this population.

## Methods

### Study design, patients and setting

We assessed the prevalence of PA and its association with respiratory disease in a prospective study including patients, aged 0–18 years, with a diagnosis of CP who attended either specialized day care centers in Flanders and/or the Antwerp Reference Centre of Cerebral Palsy in the Antwerp University Hospital. Exclusion criteria included a known presence of PA defined as a throat swab with a positive culture for PA and eradication treatment in the last month before inclusion. Inclusion was done during two periods. Informed parental consent was obtained. First inclusion was done between August 2017 and January 2018. To further expand the study, new inclusions were done from January 2020 to September 2020. Data from patient records were extracted from the electronic medical records by three reviewers.

### Sample collection and respiratory symptom questionnaire

Throat swabs were used to evaluate lower airway microorganisms. These were taken from asymptomatic patients, only one swab per patient was taken. Informed parental consent was obtained. All throat swabs taken, during the study period were reviewed, and the presence of PA and other bacteria was recorded. Follow-up of respiratory symptoms was done by the Liverpool respiratory symptom questionnaire (LRSQ) after 3 months. This questionnaire was previously validated in preschool children with wheeze, and children with cystic fibrosis (7). The LRSQ consists of eight domains. The first six domains assess respiratory symptoms during daytime, night-time, colds, interval (between colds), symptoms with activity and other symptoms. The remaining two assess the impact of symptoms on the child and family.

### Statistical analysis

Analyses were conducted using SPSS (V27).

## Results

In 2017 and 2020, 38 children were included and in 2020, 41 children were included from the outpatient CP clinic and/or specialized day-care centers. A throat swab and a completed LRSQ after 3 months were received from 79 children with CP. Demographic data, respiratory characteristics and data of comorbidities from patient records were extracted from the electronic medical records (Table 1). Feeding difficulties, malnutrition, cough efficiency, airway clearance therapy and epilepsy were statistically significant associated with GMFCS stages. Twenty-eight patients (35%) were found to have at least one positive respiratory culture. Only 4 patients (5%) were infected with PA. Gram negative bacteria were isolated in 22% of the positive throat swabs, *S. aureus* was found in 14%. Most pathogens were found in patients with higher GMFCS score (GMFCS III, IV and V) (Table 2). Results of the LRSQ showed that 52% of these patients reported having 1 cold in the past 3 months. Six patients (8.5%) had a lower respiratory tract infection. Antibiotics were prescribed in 13 of 71 patients (18%). Only three patients were admitted to the hospital, only one of these patients had a positive culture (e.g., K. oxytoca), none of these patients were colonized with PA. No statistically significant relation could be found between the number of colds and/or pneumonia, the use of antibiotics or hospitalization, and having a positive culture, colonization with *S. aureus*, *P. aeruginosa* or gram-negative bacteria.

**Table 1:** Demographic characteristics.

Patient characteristics *n* = 79		
Age (years)	Mean	8.44 (SD^a^ ± 4.74)
Gender	M: F	37: 42
Gross motor function score	Level I	19 (24.1%)
	Level II	18 (22.8%)
	Level III	9 (11.4%)
	Level IV	14 (17.7%)
	Level V	19 (24.1%)
Respiratory characteristics		Number of patients
OSA^b^		4 (5.1%)
Asthma		2 (2.5%)
Bronchiectasis		1 (1.3%)
Antibiotic prophylaxis		3 (3.8%)
Chronic respiratory medication^c^		9 (11.4%)
Chronic airway clearance therapy (e.g., Physiotherapy)		9 (11.4%)
Chronic oxygen therapy		1 (1.3%)
NIV^d^		5 (6.3%)
Comorbidities		Number of patients
GER^e^		10 (12.7%)
Feeding difficulties		34 (43%)
Malnutrition		12 (15.2%)
Scoliosis *(missing n = 1)*		7 (8.9%)
Epilepsy *(missing n = 1)*		28 (35.4%)

^a^ Standard deviation.

^b^ Obstructive sleep apnea.

^c^ Chronic respiratory medication: Salbutamol, Fluticasone propionate, Salmeterol/Fluticasone propionate, Acetylcysteine.

^d^ Non-invasive ventilation.

^e^ Gastroesophageal reflux.

**Table 2:** Descriptive statistics and chi square tests for positive culture, gram-negative bacteria, and *S. aureus*.

	Positive culture of any bacteria			Gram-negative bacteria			*S. aureus*		
	No	Yes	*P* value	No	Yes	*P* value	No	Yes	*P* value
Predictors									
GMFCS			**.016**			**.006**			**.040**
I–II	29 (36.7%)	8 (10.1%)		34 (43.0%)	3 (3.8%)		35 (44.3%)	2 (2.5%)	
III–IV–V	22 (27.8%)	20 (25.3%)		28 (35.4%)	14 (17.7%)		33 (41.8%)	9 (11.4%)	

Bold *P* value < 0.05 is considered statistically significant.

## Discussion

Although respiratory disease is an important risk factor for morbidity and mortality in children with CP, we demonstrated that the prevalence of PA in our population of children with CP without known chronic lung disease is low. Broncho alveolar lavage fluid samples are the gold standard for evaluating lower airway microorganisms, but this technique is too invasive for the present study. We decided to use throat swabs for culture, a test that is very specific in children with cystic fibrosis, with a high negative predictive value and low positive predictive value (8). It is often used in children who are not able to expectorate sputum or in young children.

In our study, colonization with PA or other bacteria in a large sample of children with CP does not seem to be a major cause of respiratory morbidity and mortality. Previous studies, including the study by Gerdung et al. showed that children with CP with a positive culture for gram-negative bacteria, and mainly PA, had more severe respiratory disease, more Paediatric Intensive Care Unit (PICU) hospitalizations, more need for mechanical ventilation, larger pleural effusions and a longer hospitalization (5). Our study showed that there is a correlation between GMFCS score and colonization with gram-negative bacteria. The short-term respiratory consequences of being colonized with these bacteria were limited in our prospective study. Because very few colds were reported, there were also few or no reported complaints such as wheezing, coughing,…. We could not demonstrate a statistically significant correlation between number of colds, pneumonia, the use of antibiotics or hospitalization with having a positive culture, colonization with *S. aureus*, *P. aeruginosa* or gram-negative bacteria. One possible explanation for this effect during the second inclusion period could be the COVID-19 pandemic with two lockdowns and therefore less exposure to pathogens due to less social contact and extensive hygienic measures, no visitors in specialized day-care centers and closed schools. Another possible explanation regarding the limited findings of PA and respiratory symptoms is that the inclusion of the two parts of the study took place in different seasons. The first part was included from August through January, and the second part was included from January through September. During the second exclusion period, the LRSQ was conducted in the spring/summer with possibly less circulation of pathogens causing respiratory tract infections. However, we chose to detect chronic colonization in children without known chronic lung disease and then seasonal variation of pathogens may have less of an impact.

As we know life expectancy in CP can improve by both preventive measurements as initiation of early therapy concerning respiratory morbidity. The prevalence of PA in this prospective study of children with CP is low, gram-negative bacteria were most commonly found. Therefore, it is recommended to repeat and expand this study since the prevalence of respiratory tract infections is again increasing in the post-covid era.

## Data Availability

The original contributions presented in the study are included in the article/Supplementary Material, further inquiries can be directed to the corresponding author.
